# High genetic diversity of common toad (*Bufo bufo*) populations under strong natural fragmentation on a Northern archipelago

**DOI:** 10.1002/ece3.1957

**Published:** 2016-02-12

**Authors:** Steffen Roth, Robert Jehle

**Affiliations:** ^1^ The Natural History CollectionsUniversity Museum of BergenBergenN‐5020Norway; ^2^ School of Environment and Life SciencesUniversity of SalfordSalfordM5 4WTUK

**Keywords:** Anurans, genetic variation, island populations, Norway

## Abstract

The last decades have shown a surge in studies focusing on the interplay between fragmented habitats, genetic variation, and conservation. In the present study, we consider the case of a temperate pond‐breeding anuran (the common toad *Bufo bufo*) inhabiting a naturally strongly fragmented habitat at the Northern fringe of the species’ range: islands offshore the Norwegian coast. A total of 475 individuals from 19 populations (three mainland populations and 16 populations on seven adjacent islands) were genetically characterized using nine microsatellite markers. As expected for a highly fragmented habitat, genetic distances between populations were high (pairwise *F*_st_ values ranging between 0.06 and 0.33), with however little differences between populations separated by ocean and populations separated by terrestrial habitat (mainland and on islands). Despite a distinct cline in genetic variation from mainland populations to peripheral islands, the study populations were characterized by overall high genetic variation, in line with effective population sizes derived from single‐sample estimators which were on average about 20 individuals. Taken together, our results reinforce the notion that spatial and temporal scales of fragmentation need to be considered when studying the interplay between landscape fragmentation and genetic erosion.

## Introduction

In ecological and evolutionary research, populations with pronounced spatial structure are often the focus of genetic investigations. Studies can be conducted across an entire species’ range, for example to provide a better understanding of the evolutionary history underlying an observed distribution (summarized in e.g. Avise 2004). At more confined spatial and temporal scales, genetic investigations can for example reveal the extent at which dispersal shapes the structure of local populations, and how human‐induced habitat fragmentation can increase population isolation (for reviews see Keyghobadi 2007; DiBattista 2008). At all spatial levels, genetic data can help to outline management measures for species under threat (e.g. Allendorf et al. 2013).

Based on theory and largely backed up with empirical data, there is a well‐established link between the standing amount of neutral genetic variation and fitness‐associated traits which can influence the ability of populations and species to persist (e.g. Saccheri et al. 1998; Spielman et al. 2004; but see also Reed 2010). Fragmented environments result in small and isolated demes subject to loss of genetic variation, as well as in a pronounced spatial distribution of genetic diversity as predicted by habitat features (e.g. Couvet 2002; Manel and Holdegger 2013; Balkenhol et al. 2015). Despite these clear causal relationships, however, it is often notoriously difficult to discern between population declines which are purely caused by habitat reduction and declines which are accelerated by genetic erosion (Bijlsma and Loeschcke 2012; Fraser et al. 2014). This poses a general problem in conservation biology, which can, at least in part, be attributed to overgeneralizations across spatial and temporal scales. Peripheral populations, for example, might be characterized by lower amounts of genetic variation for reasons other than habitat fragmentation, and low allelic diversity is not necessarily linked to fitness reductions (Ficetola et al. 2007; Eckert et al. 2008; but see also Dufresnes and Perrin 2015). A population genetic signature of habitat fragmentation also needs time to accumulate after human‐induced fragmentation occurred, leading to a genetic structure which does not necessarily match with current landscape features (Zellmer and Knowles 2009; Anderson et al. 2010; Chiucchi and Gibbs 2010; Safner et al. 2011).

The limited dispersal ability and natural population structure of pond‐breeding amphibians makes them key organisms to highlight the genetic consequences of habitat fragmentation to wild populations (Cushman 2006; Rivera‐Ortíz et al. 2015). Indeed, a range of studies has documented lower amount of genetic variation for small and isolated populations which often goes hand in hand with reduced fitness (Rowe and Beebee 2003; Johansson et al. 2007; Allentoft and O'Brien 2010; but see also Luquet et al. 2013). A particular case of spatial genetic structure relates to pond‐breeding amphibians residing on small offshore islands. Because salt water is a natural barrier to dispersal, amphibian populations within islands can form networks of potentially connected demes, whereas populations between islands over ecological timescales are isolated by non‐permeable ocean, as an assumption leading to a nested spatial population structure (Seppä and Laurila 1999; Lampert et al. 2007; Wang et al. 2014). Amphibian populations on nearby islands can further bear signatures of differential life‐histories and local adaptation, demonstrating long‐term effects of isolation (Lind and Johansson 2009; Rogell et al. 2010a,b; Lind et al. 2011; Velo‐Antón et al. 2012).

**Figure 1:**
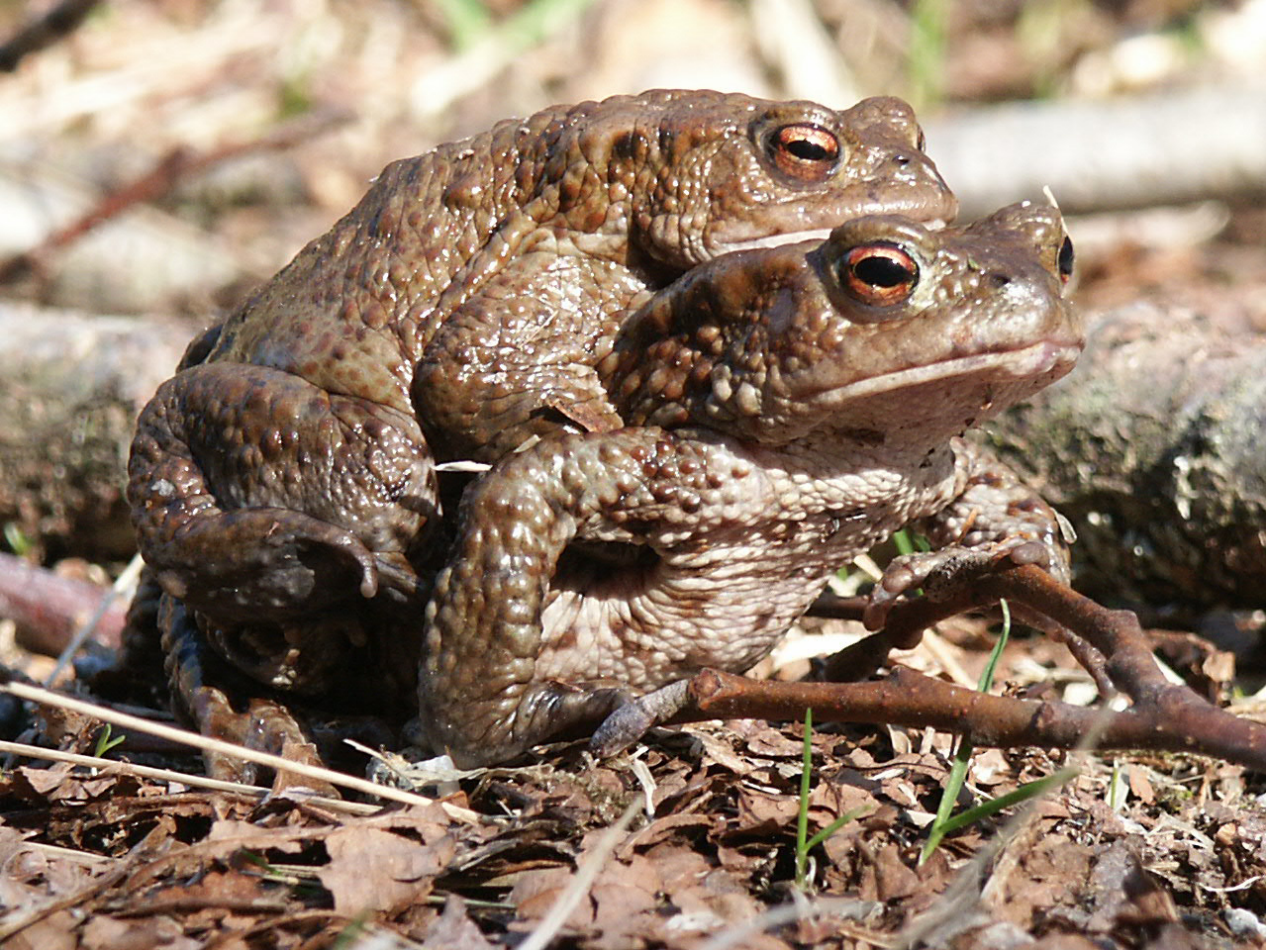
A common toad (*Bufo bufo*) pair in amplexus from the study area in Norway.

The common toad (*Bufo bufo*, Fig. 1) has a large distribution across central eastern and northern Europe (note that the taxonomy of *B. bufo* has recently been revised: Recuero et al. 2012; Arntzen et al. 2013, 2014). *Bufo bufo* is characterized by a regular occurrence at sites highly impacted by humans (despite measurable physiological consequences: Reading 2007; Janin et al. 2011; Orton et al. 2014), and was among the first amphibians for which adverse genetic effects of habitat fragmentation have been demonstrated (Hitchings and Beebee 1998). Possibly due to life history traits such as skewed sex ratios and high fecundity, *B. bufo* populations are also often characterized by low amounts of genetic variation and low effective population sizes, combined with a spatial differentiation which exceeds other co‐occurring anurans (Scribner et al. 1997; Seppä and Laurila 1999; Brede and Beebee 2004; Flavenot et al. 2015).

In the present study, we use microsatellite markers to describe the genetic structure of *B. bufo* populations on an archipelago along the Norwegian coast, with toads inhabiting adjacent islands which were separated from the mainland at least since the last glaciation about 10,000 years ago. Our main aim is to document the standing amount of neutral genetic variation under assumed long‐term isolation for a highly deme‐structured species. We demonstrate that *B. bufo* populations are able to maintain a significant amount of neutral genetic variation despite strong natural dispersal barriers between them, contributing to our understanding of the link between landscape fragmentation, population declines, and genetic erosion.

## Material and Methods

### Study sites and field work

**Figure 2:**
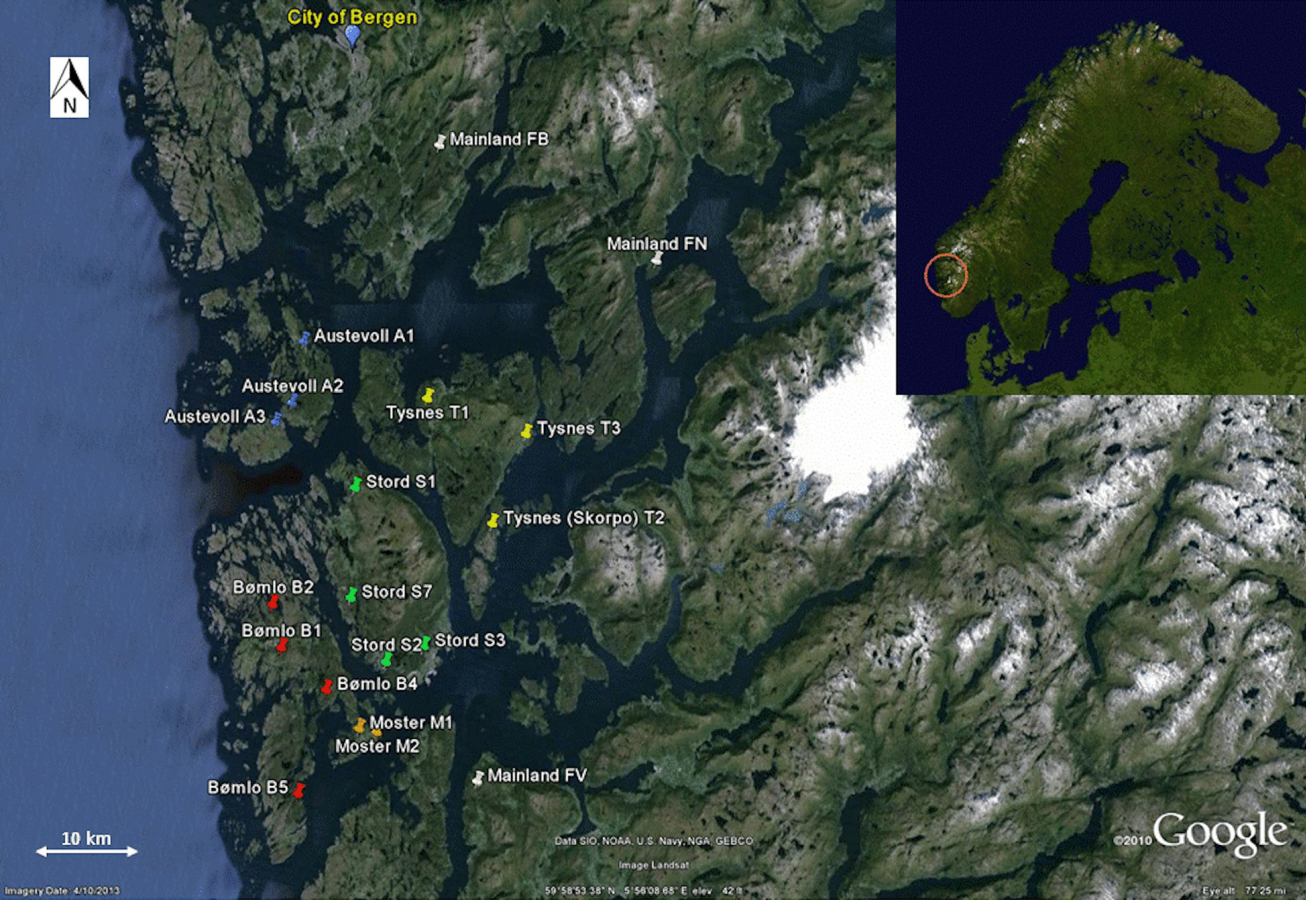
Location of 16 *Bufo bufo* study populations in Norway.

Field work was conducted in an area of approximately 30 × 35 km south of Bergen (Fig. 2), and formed part of a herpetological inventory (see Roth 2011 for more details on the study sites). The entire study area is characterized by mountainous terrain. In total, 19 *B. bufo* populations inhabiting bog tarns, ponds and small lakes were sampled during the peak of the 2008 breeding season (April–May, Table 1). Three populations (FN, FB, and FV) were situated on the mainland, and 16 populations were situated on seven offshore islands (1–4 populations per island). The islands are between 8.7 km^2^ and 238 km^2^ in size (Austevoll‐Huftarøy with populations A1 and A2: 51 km^2^; Austevoll‐Selbørn with population A3: 23.5 km^2^; Bømlo with populations B1, B2, B4, and B5: 170 km^2^; Moster with populations M1 and M2: 12 km^2^; Stord with populations S1, S2, S3 and S7: 238 km^2^; Tysnes‐Tysnesøya with populations T1 and T3: 200 km^2^; Tysnes‐Skorpo with population T2: 8.7 km^2^), and all islands are inhabited by humans. Six of these seven islands are connected with other islands or the mainland through 16–100 year old road bridges, and pairwise geographic (line‐in‐sight) distances between populations ranged between 1.20 km and 73.78 km. Although we lack historical records about the studied *B. bufo* populations we assume that they are of natural origin.

**Table 1:** Descriptive population genetic parameters across 19 *Bufo bufo* populations. Island populations are labelled with letters referring to the island of origin, and numbers based on field work. The last three populations are situated on the mainland

Population	*n*	A/L	AR	*H*_o_	*H*_e_	HW disequilibria	ML	PA
A1	15	3.67	2.59	0.51	0.55	0	1	0
A2	14	3.22	2.54	0.41	0.52	0	2	0
A3	12	2.78	2.36	0.58	0.54	0	1	2
B1	34	6.44	3.03	0.54	0.62	*Bbuf11, Bbuf62, Bbuf65*	0	3
B2	32	4.44	2.74	0.54	0.54	0	0	0
B4	34	6.22	3.24	0.56*	0.66	*Bbuf11, Bbuf54, Bbuf11*	0	4
B5	38	5.66	2.65	0.53*	0.55	*Bbuf*49, *Bbuf62, Bbuf65*	1	2
M1	27	4.89	3.55	0.60	0.65	0	0	1
M2	36	4.67	3.04	0.57*	0.60	*Bbuf13, Bbuf59, Bbuf65*	0	1
S1	15	3.22	2.80	0.61	0.54	*Bbuf*49	1	0
S2	16	2.89	2.43	0.45	0.41	0	3	1
S3	27	3.67	2.09	0.54	0.51	*Bbuf*49, *Bbuf*65	1	2
S7	46	3.89	2.47	0.55	0.58	*Bbuf*49	1	0
T1	9	3.44	2.64	0.53	0.60	0	1	0
T2	26	4.67	2.72	0.62*	0.63	*Bbuf*65	0	2
T3	5	2.78	2.90	0.57	0.54	0	1	0
FN	24	8.00	2.52	0.59	0.70	*Bbuf65*	0	6
FV	30	6.22	3.35	0.76	0.73	*Bbuf*49	0	11
FB	35	6.78	3.44	0.64*	0.69	*Bbuf*65, *Bbuf*15	0	5

*n*, number of samples genotyped; A/L, mean number of alleles per locus; AR, mean allelic richness; *H*_o_ and *H*_e_, observed and expected mean heterozygosity; HW disequilibria, loci out of HWE; *denotes overall significant deviations from neutral expectation at a Bonferroni‐corrected *P* value (0.0056); ML, number of monomorphic loci; PA, number of private alleles.

The postglacial sea level changes in the coastal area of western Norway are characterized by uplifting of land masses after the recession of glaciers (Kaland 1984; Svendsen and Mangerud 1987), and a shared shoreline history of all studied islands since the end of the last ice age suggests their similar age (see e.g. Kaland 1984). In general, all sea lines show a rapid regression between 10,000 and 8700 years B.P. from 30 m above the present sea level to *ca*. 4 m above present sea level. A transgression took place between 8500 and 7200 years B.P. when the shore level rose to *ca*. 11 m above the present level. Between 7200 and 6000 years B.P. the shore level was almost constant before a slow regression. Since all islands have peaks between 50 and 750 m above the present sea level, they exist since the last ice age without land bridges between them, at however varying size dynamics (detailed data not shown).

DNA samples were collected through dip‐netting for larvae or through collecting eggs before raising them in water‐filled containers until late egg stages, and stored in absolute ethanol. When performing the sampling, care was taken that the whole pond shore was evenly sampled and that the number of eggs per egg string was minimized; whenever possible, sites were visited several times. Long‐term monitoring of a subset of populations revealed that population size estimates based on single clutch counts are not representative for true population size (data not shown). We therefore lack good approximations of population census sizes.

### Genetic analyses

DNA extractions were performed using standard phenol–chloroform procedures (Bruford et al. 1998). Microsatellite genotypes were obtained using PCR primers described in Brede et al. (2001) to amplify nine loci (*Bbuf*11, *Bbuf*13, *Bbuf*15, *Bbuf*24, *Bbuf*46, *Bbuf*49, *Bbuf*54, *Bbuf*62, *Bbuf*65). Each 10 mL PCR contained 10–50 ng DNA, 5 pmol (5 mmol/L) of each primer, 0.15 mmol/L of each dNTP, 1.5 mmol/L MgCl_2_, and 0.5–1.0 U Taq polymerase (Advanced Biotechnologies, Columbia, MD) in the manufacturer's buffer. The PCR profiles were 94°C for 2 min, followed by 39 cycles of 94°C for 30 sec, the primer‐specific annealing temperatures as in Brede et al. (2001) for 30 s, and 72°C for 30 sec. Primers were labelled with fluorochromes, and alleles were visualized using an ABI 3730 capillary sequencer and scored with the software genemapper (Applied Biosystems, Foster City, CA, USA).

Observed (*H*_o_) and expected (*H*_e_) heterozygosities, and departures from Hardy–Weinberg equilibrium at each locus and population were computed with genepop 4.0, using the implemented Markov Chain method (10^6^ runs) to obtain unbiased estimates of Fisher's exact tests (Rousset 2008). Null allele frequencies were estimated using microchecker (van Oosterhout et al. 2004). Spatial genetic differentiation between ponds was described using pairwise *F*_st_ also using genepop 4.0, with Bonferroni corrections to give table‐wide significance levels of *P *=* *0.05. fstat (Goudet 1995) was used to obtain estimates of allelic richness based on the minimum population sample size (*n *=* *5). Isolation‐by‐distance scenarios were tested using Mantel tests to correlate *F*_st_ and log‐transformed geographic distances as implemented in the software IBDWS version 3.23 (Bohonak 2002).

The nested sampling regime (several populations per island, with several islands under consideration) allowed to discern between within‐ and between‐island differentiation using analysis of molecular variance (AMOVA) as implemented in arlequin (Excoffier and Lischer 2010). We considered four alternative scenarios of population groupings (all islands as groups and all mainland populations as individual groups; island populations only with all islands as groups; all islands as groups and all mainland populations as a single group; island and mainland populations as two groups). Because missing data influence the results, a locus‐by‐locus AMOVA was used to adjust the sample sizes for each locus and the point estimators of variance components to estimate *F*‐statistics more accurately (Excoffier et al. 1992). The hierarchical population structure was further investigated using the algorithm implemented in BAPS 6.0 (Cheng et al. 2013). This approach enables us to distinguish an enforced substructure (in our case, defined on the basis of ponds) from a potentially more meaningful structure reflected in the data set (such as for example all ponds on one island). The criteria used to separate populations are based on whether any population pair in the sample can be regarded as a single population (for details see Corander et al. 2003). Posterior distributions are derived from an MCMC algorithm (we considered 500,000 runs after 100,000 burn‐ins), and we set a lower probability bound of 0.05 for partitions to be considered in a final model.

Two single‐sample measures of effective population size (*N*_e_) were obtained. We used the linkage disequilibrium approach (Waples 2006) as implemented in neestimator 2.0 (Do et al. 2014), and the sibship method as implemented in colony2 (Wang 2009). We specified genotype error rates as calculated with microchecker, assumed a polygamous breeding system for females but not for males (Sztatecsny et al. 2006) and used the full likelihood model with medium precision and no prior information.

## Results

In total, we genotyped 475 individuals across the 19 study populations, with an average of 25 samples per population (range: 5–46); the overall PCR success rates across genotyped loci was 78%. The analysis conducted in microchecker revealed evidence for null alleles in 5/19 populations for locus *Bbuf*65 (populations B4, FB, FN, T1, T2), 2/19 populations for locus *Bbuf*15, and 1/19 populations for locus *Bbuf*54; all other loci and populations revealed no evidence for null alleles or large allele dropouts (detailed data not shown). Given the geographic location close to the northern border of the species’ geographic range as well as the assumed ecological isolation, the studied populations are characterized by moderate to high overall genetic variation (between 2.78 and 8.00 alleles per locus and population, Table 1). Five of 19 populations are characterized by heterozygote deficiencies at specific loci. Nine of 16 island populations contained up to three monomorphic loci, whereas all loci were polymorphic in the mainland populations. The three mainland populations were characterized by higher overall measures of genetic variation than island populations, bearing 25 alleles (17.2% of the overall allelic diversity represented with 145 alleles across loci) which were absent on islands as well as high overall levels of allelic richness (Table 1).

**Figure 3:**
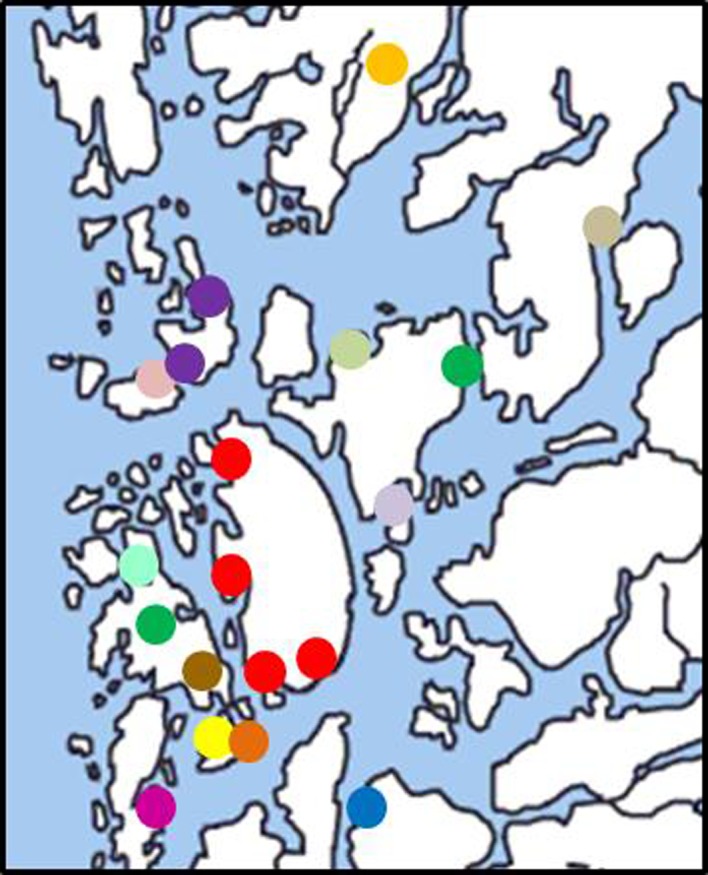
Most likely partition of genetic units (*P *=* *0.84) represented by different colours as identified by the algorithm implemented in the software BAPS. For more details see text.

As expected from the geographic setting, the populations are characterized by high genetic differentiation. Pairwise *F*_st_ values were significant for all but three comparisons which involved population T3 for which only five samples were available (T3‐B1, T3‐B4, T3‐FN, Table 2). Remarkably, there were no marked differences in pairwise *F*_st_ between populations separated by terrestrial habitat and populations separated by salt water. The isolation‐by‐distance analysis revealed no relation between log geographic distance and *F*_st_ (the regression coefficient was even slightly negative; *Z* = −3919.53, *r* = −0.02, *P* = 0.56). The analyses of molecular variance (AMOVA) revealed similar patterns for the four scenarios of population grouping (Table 3). The variance among populations always exceeded the variance among islands or between islands and the mainland, demonstrating that ponds are the most important structural units to define spatial population structure; island populations as a whole were not more differentiated from mainland populations than within each other. The high amount of genetic differentiation is also reflected in the analysis as implemented in BAPS, which regarded the 19 populations as 14 independent clusters (Fig. 3). Whenever several populations were merged into a single cluster, they largely comprised all populations from one island. One cluster comprised all populations from Stord (S1, S2, S3, and S7), and one cluster comprised two populations from Austevoll‐Huftarøy (A1 and A2). A further cluster contained one population from Bømlo (B1) and one population from Tysnes (T3) without geographic proximity, a result which is however likely due to the low sample size for this population (Table 1).

**Table 2:**
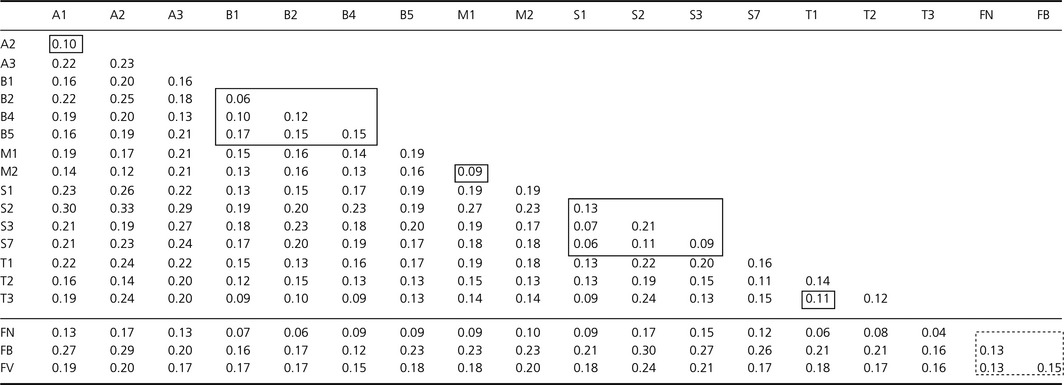
Pairwise *F*_st_ values between *Bufo bufo* populations. Comparisons between populations on the same island are shown in boxes, comparisons between mainland populations are shown in a dashed box. The three mainland populations are below the horizontal line

**Table 3:** Analysis of molecular variance (AMOVA) between island and mainland *Bufo bufo* populations using four alternative groupings

Structure tested	Sums of squares	Variance components	Percentage variation
All mainland vs. all island populations			
Among groups	42.4	0.18	2.4
Among populations within groups	373.6	0.51	15.7
Within populations	1913.9	2.70	81.9
All islands as groups, all mainland populations as one single group			
Among groups	225.8	0.20	6.1
Among populations within groups	190.3	0.37	11.3
Within populations	1913.9	2.70	82.6
All islands as groups, all mainland populations as individual groups			
Among groups	275.1	0.23	7.0
Among populations within groups	140.9	0.34	10.4
Within populations	1913.9	2.70	82.7
Island populations only, islands as groups			
Among groups	176.3	0.30	6.5
Among populations within groups	147.9	0.36	11.4
Within populations	1514.0	2.60	82.1

The estimates of *N*_e_ largely converged between the linkage disequilibrium method and the sibship method, but the latter was characterized by markedly lower confidence limits (Table 4). Typical effective population size values were in the order of 20 individuals.

**Table 4:** Single sample genetic measures of effective population size for 19 *Bufo bufo* populations. For more details see text

Population	Linkage disequilibrium method	Sibship method
A1	40.4 (5.1–∞)	38 (16–223)
A2	39.2 (2.6–∞)	17 (8–40)
A3	2780.6 (2.0–∞)	26 (12–114)
B1	24.7 (14.2–54.8)	36 (22–64)
B2	30.8 (12.0–∞)	28 (18–48)
B4	20.1 (11.3–45.7)	31 (18–55)
B5	23.7 (12.2–68.0)	19 (11–38)
M1	29.4 (12.2–2113.3)	27 (16–52)
M2	6.3 (15–∞)	3 (1–31)
S1	234.4 (6.8–∞)	25 (12–77)
S2	12.3 (1.6–∞)	12 (6–30)
S3	24.5 (8.1–∞)	21 (10–60)
S7	808.4 (27.3–∞)	24 (14–42)
T1	15.1 (9.5–∞)	48 (18–∞)
T2	101.9 (19.7–∞)	13 (7–30)
T3	5.0 (1.7–∞)	40 (6–∞)
FN	91.0 (26.4–∞)	48 (29–90)
FB	65.7 (21.8–∞)	34 (21–61)
FV	89.6 (22.4–∞)	18 (10–35)

## Discussion

The main findings from our study on genetic variation of anuran populations strongly subdivided across a Northern European archipelago are twofold. Firstly, we document a pattern of pronounced spatial genetic variation which largely reflects the geographic setting. Population differentiation was overall high; remarkably, the genetic signature of individual populations exceeded the signature of population clusters on specific islands. Secondly, although genetic variation was lower at island populations compared to mainland populations, we reveal an overall rather high amount of neutral genetic variation despite putative long‐term population isolation. We use our findings to shed further light on the link between genetic variation and landscape fragmentation.

Genetic differentiation is generally shaped by the interplay between demographic history, isolation and drift (e.g. Marko and Hart 2011). As expected from populations which are ecologically isolated from each other, the measured *F*_st_ values were above the values previously reported for common toads (Brede and Beebee 2004; Wilkinson et al. 2007; Martínez‐Solano and Gonzalez 2008; Luquet et al. 2015; see also Seppä and Laurila 1999 for a study based on allozymes). That 10 of 16 island populations and none of the three mainland populations possessed at least one monomorphic locus polymorphic elsewhere in the study area confirms that islands were likely colonized from the mainland, and also provide evidence for higher genetic drift in more peripheral populations. Genetic connectivity measures between amphibian populations are distinctly scale‐dependent, with the grain of investigation determining for example whether a regular exchange of individuals is governed by demography‐driven metapopulation processes, or whether specific landscape elements act as corridors or barriers for dispersal (Jehle et al. 2005; Anderson et al. 2010; Angelone et al. 2011; Metzger et al. 2015). The geographic distances between our populations on specific islands largely exceeded the migration distances documented for common toads (e.g. Sztatecsny and Schabetsberger 2005). However, as our sampling was rather opportunistic we cannot exclude that unsampled ponds served as stepping stones for inter‐pond dispersal. Given the high *F*_st_ values between our study populations, we however rather discard this as a shaping force to determine the population structure we observe.

The algorithm implemented in the software BAPS failed to find conclusive evidence for connectivity between populations isolated by salt water (see also e.g. Martínez‐Solano and Lawson 2009). Nevertheless, we do not fully exclude rare events of dispersal across islands or from the mainland to islands, for example through human‐aided stowaways on vehicles and boats or drift wood rafting (as reported elsewhere, Measey et al. 2007; White and Shine 2009). A main result of the present study was that terrestrial habitat also appears to represent a significant barrier to migration, as *F*_st_ values between populations separated by only terrestrial habitat were substantial, both on islands as well as on the mainland. This is likely caused by the rather mountainous topology as well as fragmentation by fjords (but see Sztatecsny and Schabetsberger 2005 who demonstrate that common toads can cover significant altitudinal differences), in combination with high philopatry of *B. bufo* which promotes higher genetic differentiation than is observed for other anurans (Brede and Beebee 2004; Flavenot et al. 2015).

In line with the expectation that island populations were founded by colonization from the mainland, they were characterized by lower levels of genetic variation. However, despite a notable effect of isolation on genetic diversity we encountered an overall standing amount of genetic variation which is comparable to previous studies on *B. bufo* elsewhere in Europe (Brede and Beebee 2004; Wilkinson et al. 2007; Martínez‐Solano and Gonzalez 2008). While high neutral genetic variation for otherwise ecologically isolated amphibian populations has previously been documented (Kraaijeveld‐Smit et al. 2005; Tolley et al. 2010), we did expect less genetic diversity at the northern periphery of the species’ range. How can a high standing amount of genetic variation on islands be explained? For island populations with known founder history, high levels of maintained heterozygosity have previously been attributed to selection (Kaeuffer et al. 2007). Common toads have high mortality rates prior to reaching adulthood, for example suggesting that selection against inbred and/or more homozygote individuals can operate before reproduction. A population‐wide increase in average heterozygosity between early eggs or tadpoles and later stages has indeed been previously documented in other anurans (Ficoleta et al. 2011; Ursprung et al. 2012). It is nevertheless remarkable that we did not find the drastically reduced levels of genetic variation as documented for a similar species (the natterjack toad *Epidalea calamita*) on rocky outcrop islands also along the Scandinavian coast (Rogell et al. 2010c; see also Höglund et al. 2015 for a non‐neutral gene). While our study area comprised a similar geographic scale, the studied common toad populations were mostly on larger and less peripheral islands than the studied natterjack toads (see also Rogell et al. 2010a,b). An earlier study on *B. bufo* based on allozymes (Seppä and Laurila 1999) also found no effect of geographic isolation on genetic variation.

The obtained estimates of *N*_e_ were rather high, given the male‐biased sex ratios of *B. bufo* populations and *N*_e_ (*N*_b_) estimates obtained previously (Scribner et al. 1997); nevertheless they were within the lower range of comparative values across 90 populations encompassing four ranid species (Phillipsen et al. 2011). In line with population genetic theory, high *N*_e_ values for amphibian populations are usually accompanied by high standing amounts of genetic variation (Beebee 2009; Phillipsen et al. 2011), which we also observed in our study system. Previous studies have provided evidence for high *N*_e_ values in comparison to population census sizes when census sizes are low (genetic compensation, Jehle et al. 2005; Beebee 2009). Our *N*_e_ were rather similar across populations, supporting that genetic compensation can also act for *B. bufo*; we however lack detailed population census size data to further investigate this assumption. The genetic mating system of *B. bufo* is expected to depend on sex ratios and population densities (Sztatecsny et al. 2006), and it is possible that typical *N*_e_ values for distal populations such as on offshore islands can be higher relative to census sizes than for core populations. Despite islands having a long history as natural laboratories for evolutionary studies, we still miss a comparative investigation which compares *N*_e_/*N* values between island and mainland populations.

What do our inferences tell us about conservation concerns for populations in landscapes recently fragmented by humans? We demonstrate that amphibian populations are able to maintain significant levels of genetic variation in naturally strongly fragmented landscapes, despite clear genetic effects of fragmentation though high differentiation. While genetic erosion in naturally small populations is expected to accumulate over time, it can be compensated for when the environment is stable (e.g. Kaeuffer et al. 2007; Charlier et al. 2012). It also has been demonstrated for another amphibian that increased fragmentation through population loss does not necessarily predict the degree of spatial genetic structure (Tobler et al. 2013). Taken together, we reinforce the notion that timing of fragmentation relative to the pace of possible negative genetic consequences (ultimately governed by the evolutionary potential of populations) is crucial to predict whether landscape fragmentation result in the loss of genetic variation (Anderson et al. 2010). Although links between isolation and adaptive genetic variation as well as fitness‐related traits were beyond the scope of the present study, our findings further suggest that amphibian populations are able to thrive under scenarios of high fragmentation given they have sufficient time to adapt.

## Conflict of Interest

None declared.
